# End-of-life care in German and Dutch nursing homes: a cross-sectional study on nursing home staff’s perspective in 2022

**DOI:** 10.1186/s13690-024-01316-2

**Published:** 2024-06-14

**Authors:** Ann-Kathrin Bauer, Alexander Maximilian Fassmer, Sytse U. Zuidema, Sarah I. M. Janus, Falk Hoffmann

**Affiliations:** 1 Department of Health Service Research, Carl von Ossietzky Universität Oldenburg, Oldenburg, Germany; 2 Institute of Medical Genetics, Carl von Ossietzky Universität Oldenburg, Oldenburg, Germany; 3 Department of Primary and Long-Term Care, University of Groningen, University Medical Center Groningen, Groningen, The Netherlands

**Keywords:** End-of-life care, Nursing home residents, Germany, The Netherlands, Nursing staff

## Abstract

**Background:**

As society ages, the need for nursing home care is steadily increasing and end-of-life care of nursing home residents has become increasingly more important. End-of-life care differs between Germany and the neighbouring Netherlands. For example, a much higher proportion of German compared to Dutch nursing home residents is hospitalized at the end of life. Therefore, the aim of this study was to evaluate end-of-life care in German and Dutch nursing homes.

**Methods:**

In this cross-sectional study, a postal survey was sent to 600 randomly selected German and Dutch nursing homes each and addressed to the nursing staff management. Participants were asked to estimate the percentage of nursing home residents whose wishes for emergency situations (e.g. cardiopulmonary resuscitation) are known and to indicate whether facilities offer advanced care planning (ACP). They were also asked to estimate whether general practitioners (GPs)/elder care physicians (ECPs) and nursing home staff are usually well trained for end-of-life care. Finally, participants were asked to estimate the proportion of nursing home residents who die in hospital rather than in the nursing home and to rate overall end-of-life care provision.

**Results:**

A total of 301 questionnaires were included in the analysis; 199 from German and 102 from Dutch nursing homes (response 33.2% and 17.0%). German participants estimated that 20.5% of residents die in the hospital in contrast to the Dutch estimation of 5.9%. In German nursing homes, ACP is offered less often (39.2% in Germany, 75.0% in the Netherlands) and significantly fewer wishes for emergency situations of residents were known than in Dutch nursing homes. GPs were considered less well-trained for end-of-life care in Germany. The most important measures to improve end-of-life care were comparable in both countries.

**Conclusion:**

Differences in (the delivery and knowledge of) end-of-life care between Germany and the Netherlands could be observed in this study. These could be due to structural differences (ECPs available 24/7 in the majority of Dutch nursing homes) and cultural differences (more discussion on quality of life versus life-sustaining treatments in the Netherlands). Due to these differences, a country-specific approach is necessary to improve end-of-life care.

**Supplementary Information:**

The online version contains supplementary material available at 10.1186/s13690-024-01316-2.

**Table:** 

Text box 1. Contributions to the literature
• There are several under-researched differences in the end-of-life care of nursing home residents in Germany and the Netherlands.
• This study found that in the Netherlands, fewer residents were expected to die in the hospital, more nursing homes offered ACP and more residents’ wishes for emergency situations were known.
• Structural and cultural differences between the two countries could contribute to these discrepancies.
• Overall, more and better-qualified nursing staff and better integration of palliative care would improve the quality of end-of-life care.

## Background

In an increasingly ageing society, the need for nursing home places is growing [1]. Nursing home residents often have multiple chronic diseases. Usually, they spend their last phase of life in nursing homes [2, 3]. Therefore, dignified and appropriate end-of-life care in nursing homes has become considerably more important in recent years [4].

However, nursing home residents are frequently transferred to the hospital during their last phase of life [5]. Hospitalization at the end of life is associated with great stress for the person concerned and their relatives and can also be associated with other negative health consequences such as functional and mental decline, delirium, and nosocomial infections [6]. When considering the available studies worldwide, significant variations in hospitalization in the last month of life of nursing home residents with and without dementia have been described [5, 7]. In Germany, hospitalization in the last month of life occurs in up to half of all nursing home residents [8]. In comparison, in the neighbouring Netherlands, only about 8% of nursing home residents are hospitalized in the last month of life [9]. It is estimated that in Germany around 30% of nursing home residents die in the hospital whereas in the Netherlands this is true for about 6% [8, 10].

However, a large proportion of these hospitalizations are potentially avoidable [11]. A valuable tool that residents can use to ensure that their end-of-life wishes are known is advanced care planning (ACP) [12]. ACP allows residents to make independent decisions about their medical treatment and helps caregivers tailor care to residents' individual preferences [13]. These decisions can be recorded in advance directives, [14] which are statements that enable a person’s autonomy by giving directions for future care decisions [15]. In Germany, for about half of nursing home residents (45.9% to 47%) an advance directive is available [16, 17]. In the Netherlands, ACP and the importance of documentation of end-of-life decisions are increasingly recognized. In 2007–2011 only 4.9% of Dutch nursing home residents had an advanced directive [9]. In comparison, in 2015–2016, ACP was officially in place in 33% of older people (general population) in the Netherlands [18]. In Dutch nursing homes, care goals for resuscitation and hospitalization are increasingly documented in physician treatment orders (PTOs). In the most recent study, 82% of the nursing home residents had a PTO [19].

These discrepancies in hospitalization and documented care wishes between the two countries point towards fundamental differences in end-of-life care of nursing home residents. However, so far, studies are not well comparable due to difference in methodology [20].

Therefore, the aim of this cross-national study was to evaluate end-of-life care in German and Dutch nursing homes.

## Methods

### Study design and data collection

This cross-sectional study was embedded in the sub-project "Medical care provision in nursing homes and its influence on residents’ health" of the study CHARE-GD I (Comparison of healthcare structures, processes and outcomes in the Northern German and Dutch cross-border region I) [21]. Data were collected through a postal survey sent to 600 each randomly selected German and Dutch nursing homes. As a source population, all 11,409 German nursing homes were identified by the Care Navigator, which is offered by the Federal Association of Local Health Insurance Funds ("AOK Pflegenavigator"). The 1810 Dutch nursing homes were identified by Caremap of the Netherlands ("Zorgkaart Nederland"), an initiative of the Dutch patient federation (patiëntenfederatie Nederland). The listed facilities were manually checked. Type 1 (verpleeghuizen) and type 2 (verzorgingshuizen) nursing homes were included as Zorgkaart Nederland does not differentiate between them. In Type 2 nursing homes medical care of residents is usually provided by general practitioners (GPs), as it is the case for German nursing homes. In Type 1 nursing homes, medical care is provided by specialized elder care physicians (ECPs) who are usually available around the clock [20, 22, 23].

The questionnaire (see supplementary file 1) was sent to each selected nursing home in May 2022, preferably addressed to the nursing staff manager if the name was available through manual search. If the name of the home's director or executive board was known instead, it was used. Only if no contact person could be found, the questionnaire was addressed to the current nursing staff management (without personal salutation). A reminder was sent after three weeks. Participants had the option of completing the questionnaire on paper or digitally using a web link/QR code from the study information letter. All data were collected anonymously. The data from the questionnaires that were returned by post were entered into the database by one researcher and validated by a second researcher. Unclear answers were discussed with a third and fourth researcher.

The study received waivers by the local medical ethics committee of the Carl von Ossietzky University of Oldenburg (No. 2022–012) and from the medical ethics review board of the University Medical Center Groningen in the Netherlands (2022/035).

### Questionnaire

The four-page questionnaire was developed by an interdisciplinary team from Germany and the Netherlands based on the previous HOMERN study [16]. The questionnaire consisted of four parts comprising medical care provision in nursing homes (I), hospital transfers (II), end-of-life care (III), and facility and residents’ characteristics (IV). This study focuses on end-of-life care. In this part, participants were asked to estimate the percentage of nursing home residents whose wishes for emergency situations are known with the questions “Please estimate: For which percentage of your residents are the care wishes for the following emergency situations known? Cardiopulmonary resuscitation (CPR), invasive (tube) ventilation, treatment in intensive care unit, hospital transfer” (see supplementary file 1, question 9). They were also asked to indicate whether facilities offer ACP with the question “Does your facility offer advanced care planning (ACP)?” (supplementary file 1, question 10). They were asked to rate communication with relatives regarding end-of-life on a 4-point Likert scale with the question “How easy is it for you or the nursing staff in your facility to talk to relatives of your residents about the end of life?”. The answer options ranged from very easy to very difficult (supplementary file 1, question 11). Participants were also asked to estimate on a 5-point Likert scale whether (a) GPs/ECPs and (b) nursing home staff are usually well trained for end-of-life care with the following questions: “Please assess the overall care situation in Germany/ the Netherlands: (a) General practitioners are generally well trained to provide end-of-life care for nursing home residents. (b) Nursing home staff are generally well trained in end-of-life care”. The answer options ranged from “0 = totally disagree” to “4 = totally agree” (supplementary file 1, question 12). Furthermore, participants were asked to estimate the proportion of nursing home residents who die in hospital with the question “How high is the proportion of nursing home residents who die in hospital and not in the nursing home?”. Finally, they were asked to rate end-of-life care provision overall with the question “How is the overall standard of care provision for nursing home residents at the end of life?” The answer options were “rather poor” and “rather good”. Participants who answered "rather poor" had the opportunity to comment on potential improvement by answering the question “In your opinion, what would be the most important measure that could improve care?” (free text) (supplementary file 1, question 12).

In addition, characteristics of the respondents and nursing homes such as gender, age, position in the nursing home and duration in current professional position were surveyed. They were asked to provide information about the nursing home's sponsorship, number of beds, location (rural ≤ 20,000; semi-urban between > 20,000 and ≤ 100,000; urban > 100,000 inhabitants) and distance to the nearest hospital with an emergency department.

### Statistical analysis

Frequencies were calculated for categorical data. For continuous data, mean with standard deviation (SD) and median with interquartile range (IQR) were calculated. For the question on communication with relatives, the two answers "very easy" and "rather easy" as well as "very difficult" and "rather difficult" were combined into one item, respectively. Answers of the questions using a 5-point Likert scale were regrouped by combining 0–1 to "disagree", 2 to "neutral" and 3–4 to "agree". Differences between German and Dutch nursing homes were assessed using chi-square test (χ2-Test) and Mann–Whitney U test. The free-text responses were classified into categories that were based on a previous study [16] with slight modifications. Categories were assigned by one researcher and then independently validated by a second researcher.

Statistical analysis was conducted using IBM SPSS Statistics version 28.0.1.0 (Armonk, NY: IBM Corp.) and SAS 9.4 (SAS Institute Inc., Cary, NC, USA).

## Results

### Characteristics of respondents and nursing homes

A total of 301 questionnaires were included in the analysis; 199 from German and 102 from Dutch nursing homes (response 33.2% and 17.0%). The characteristics of the respondents and the nursing homes are summarised in Table 1. Respondents were predominantly female (79.4% in Germany and 85.9% in the Netherlands) and the majority of the questionnaires were answered by nursing staff managers (70.4% in Germany and 45.0% in the Netherlands). While the proportion of German nursing homes was higher in rural areas (≤ 20,000 inhabitants) than in the Netherlands (43.7% vs. 34.3%), the proportion in urban areas (> 100,000 inhabitants) was almost identical in both countries (22.1% vs. 22.2%). The mean distance to the nearest hospital with an emergency department was 8.7 km (SD 6.9) for German and 10.4 km (SD 8.6) for Dutch nursing homes.

**Table 1:** Characteristics of respondents and nursing homes answering the questionnaire about nursing home care in Germany and the Netherlands (data from the 2022 CHARE-GD I study)

	German nursing homes (*N* = 199)	Dutch nursing homes (*N* = 102)
**Gender**	(*n* = 199)^a^	(*n* = 99)^a^
Male	41 (20.6%)	13 (13.1%)
Female	158 (79.4%)	85 (85.9%)
Diverse	-	1 (1.0%)
**Age in years**	(*n* = 193)^a^	(*n* = 99)^a^
Mean (SD)	48.1 (10.1)	44.8 (11.9)
Median (IQR)	50.0 (40.0–56.0)	48.0 (34.0–54.0)
≤ 49	93 (48.2%)	55 (55.6%)
50–59	66 (34.2%)	35 (35.4%)
≥ 60	34 (17.6%)	9 (9.1%)
**Position in the nursing home** ^b^	(*n* = 199)^a^	(*n* = 100)^a^
Nursing staff manager	140 (70.4%)	45 (45.0%)
Facility administration	58 (29.2%)	26 (26.0%)
Other (e.g. executive board, quality management, ward management, nurses)	19 (9.5%)	34 (34.0%)
**Work experience in current position in years**	(*n* = 195)^a^	(*n* = 97)^a^
Mean (SD)	9.6 (8.4)	9.0 (9.0)
Median (IQR)	7.0 (3.0–15.0)	5.0 (3.0–15.0)
**Sponsorship**	(*n* = 194)^a^	(*n* = 92)^a^
Non-profit	110 (56.7%)	84 (91.3%)
Private	67 (34.5%)	8 (8.7%)
Public	17 (8.8%)	-
**Number of beds**	(*n* = 199)^a^	(*n* = 100)^a^
Mean (SD)	83.4 (42.3)	85.0 (75.5)
Median (IQR)	80.0 (57.0–102.0)	62.0 (33.5–112.0)
**Location**	(*n* = 199)	(*n* = 99)
Rural (≤ 20,000 inhabitants)	87 (43.7%)	34 (34.3%)
Semiurban (> 20,000- ≤ 100,000 inhabitants)	68 (34.2%)	43 (43.4%)
Urban (> 100,000 inhabitants)	44 (22.1%)	22 (22.2%)
**Distance to next hospital with an emergency department [km]**	(*n* = 197)	(*n* = 100)
Mean (SD)	8.7 (6.9)	10.4 (8.6)
Median (IQR)	7.0 (14.0–3.0)	9.0 (15.0–4.0)

*SD* standard deviation, *IQR* Interquartile range

^a^numbers differ because of missing values

^b^Multiple answers possible

- not applicable

### Advanced care planning

Overall, it was estimated that in German nursing homes significantly less wishes for emergency situations of nursing home residents were known than in Dutch nursing homes (Table 2). Respondents from German nursing homes estimated that of around half of nursing home residents wishes were known for CPR (52.8%). In Dutch nursing homes these estimates were significantly higher with wishes known for 72.5% in case of CPR (*p* < 0.0001). Additionally, significantly less wishes for hospital transfers were known for participants from German than Dutch nursing homes (55.8% vs. 72.9%; *p* < 0.0001). Significantly less German nursing homes offered ACP than Dutch facilities (39.2% vs. 75.0%; *p* < 0.0001).

**Table 2:** Nursing home staffs perceptions on end-of-life care in German and Dutch nursing homes (data from the 2022 CHARE-GD I study)

	**German nursing homes (** ***N*** ** = 199)**	**Dutch nursing homes (** ***N*** ** = 102)**	***p*** **-value**
**Estimate of care wishes known of residents in case of**			
Cardiopulmonary resuscitation	(*n* = 176)^a^	(*n* = 98)^a^	
Mean (SD)	52.8% (35.8)	72.5% (41.9)	< 0.0001^1^
Invasive (tube) ventilation	(*n* = 172)^a^	(*n* = 95)^a^	
Mean (SD)	44.7% (38.1)	60.1% (45.2)	0.0005^1^
Treatment in intensive care unit	(*n* = 177)^a^	(*n* = 95)^a^	
Mean (SD)	44.9% (36.5)	64.6% (43.2)	< 0.0001^1^
Hospital transfers	(*n* = 175)^a^	(*n* = 96)^a^	
Mean (SD)	55.8% (33.6)	72.8% (36.2)	< 0.0001^1^
**Nursing home offers advanced care planning**	(*n* = 189)^a^	(*n* = 96)^a^	
Yes	74 (39.2%)	72 (75.0%)	< 0.0001^2^
**Estimated proportion of residents dying in hospital**	(*n* = 191)^a^	(*n* = 95)^a^	
Mean (SD)	20.5% (20.0)	5.9% (15.2)	< 0.0001^1^
**Overall rating of end-of-life care in nursing homes**	(*n* = 195)^a^	(*n* = 99)^a^	
Rather poor	43 (22.1%)	9 (9.1%)	0.0058^2^
Rather good	152 (77.9%)	90 (90.9%)	
**Nursing home staff perception on end-of-life talk with relatives**	(*n* = 199)^a^	(*n* = 99)^a^	
Easy	160 (80.4%)	84 (84.8%)	0.3479^2^
Difficult	39 (19.6%)	15 (15.2%)	

*SD* standard deviation

^a^numbers differ because of missing values

^1^Mann-Whitney U test

^2^Chi-Square test

### End-of-life care

**Fig. 1:**
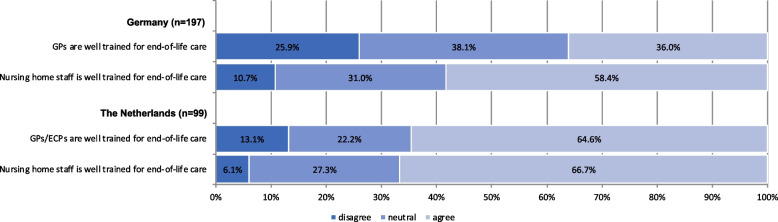
Respondents perceptions on whether general practitioners (GPs)/elder care physicians (ECPs) and nursing home staff are well trained in end-of-life care in % in Germany and the Netherlands (data from the 2022 CHARE-GD I study)

Differences in end-of-life care between German and Dutch nursing homes were observed for the perceived training of GPs/ECPs in end-of-life care, proportion of residents dying in the hospital and overall rating of end-of-life care in nursing homes (Fig. 1 and Table 2). Of German respondents, the most common response on GPs training in end-of-life care was neutral (38.1%) whereas Dutch respondents indicated that GPs/ECPs (64.6%) are well trained in end-of-life care (Fig. 1). Therefore, GPs are significantly less likely to be perceived as well trained for end-of-life care in Germany than in the Netherlands (*p* = 0.0001). There was no significant difference in the perception of nurses' training for end-of-life care between Germany and the Netherlands (*p* = 0.2765) and their perception on end-of life talk with relatives (*p* = 0.3479).

Furthermore, German respondents estimated that 20.5% (SD 20.0) of residents died in hospital rather than in the nursing home. In Dutch nursing homes, this proportion was significantly lower, with an estimate of 5.9% (SD 15.2) of residents dying in hospital (*p* < 0.0001) (Table 2). Fewer German respondents rated end of-life care as rather good than Dutch respondents (77.9% vs. 90.9%; *p* = 0.0058).

### Measures to improve end-of-life care

In total, 60 respondents commented via free text on how end-of-life care could be improved. Of these, 51 (out of 199) were German and 9 (out of 99) were Dutch. Since a person's answer could be divided into several categories, a total of 78 German comments and 14 Dutch comments were counted. The most important measures were more nursing staff (including more time per resident) (38.5% of German and 21.4% of Dutch respondents), better qualification of nursing staff (16.7% of German respondents, 14.3% of Dutch respondents) and better integration and availability of palliative care in nursing homes (9.0% of German respondents and 35.7% of Dutch respondents) (Table 3).

**Table 3:** Measures suggested by nursing home staff to improve end-of-life care in German and Dutch nursing homes (*n* = 92) (data from the 2022 CHARE-GD I study)

	**German nursing homes (** ***N*** ** = 78)**	**Dutch nursing homes (** ***N*** ** = 14)**
More nursing staff (incl. more time per resident)	38.5%	21.4%
Better qualification of nursing staff (incl. training in pall. care)	16.7%	14.3%
Better integration and better availability of palliative care in NHs	9.0%	35.7%
Better documentation and consideration of the residents will	6.4%	7.1%
Change in awareness and de-tabooing of end-of-life	6.4%	-
Closer involvement of relatives	5.1%	7.1%
Closer involvement of GPs and other medical specialists	3.8%	-
Closer involvement of other staff (volunteers, psychosocial counselling)	3.8%	-
Better qualification of the general practitioners (incl. Training in palliative care)	2.6%	7.1%
Better availability of medication (esp. Regarding pain medication)	2.6%	7.1%
Better communication and cooperation	2.6%	-
Other	2.6%	-

## Discussion

In this cross-sectional study we found that German participants estimated that 20.5% of residents die in the hospital in contrast to the Dutch estimation of 5.9%. In German nursing homes ACP is offered less often and significantly fewer wishes for emergency situations of residents were known than in Dutch nursing homes. GPs were considered less well trained for end-of-life care in Germany. The most important measures to improve end-of-life care were comparable in both countries.

### Hospitalization and advanced care planning

In our study, we found a lower in-hospital death of German nursing home residents with 20.5% compared to previous German studies. There, almost 30% of residents died in the hospital [5, 8, 16]. One study was comparable in methodology but already older, suggesting that there might be a decline in in-hospital deaths of German nursing home residents [16]. For Dutch nursing homes, our 5.9% of in-hospital deaths are in line with the previously reported 6% of residents who die in the hospital [10]. Worldwide numbers for in-hospital death of nursing home residents range from 5.9%-77.1%, showing that the Netherlands has one of the lowest percentages of hospitalization at the end-of-life [5]. German in-hospital deaths in the aforementioned study were around 30%, so above the worldwide median of 22.6% [5]. In our study, German in-hospital deaths were comparable to the worldwide median.

These differences in hospitalization in the two neighbouring countries could be due to structural differences in nursing home care. In Germany usually, several GPs are responsible for the medical care of nursing home residents. It has been shown, that on average 8.6 different GPs are responsible for one nursing home [24]. In Dutch type 1 nursing homes, which are the majority of facilities, ECPs are responsible. These are medical specialists with a specific focus on elder care medicine enabling them to do more diagnosis in the nursing home, which could prevent some hospital transports. Furthermore, ACP is part of their training [20, 23, 25].

ACP is a valuable tool to decrease hospitalization at the end-of-life [12]. In our study, 39.2% of German nursing homes offered ACP, which is comparable, but a bit less than seen in previous studies [17]. Of the Dutch nursing homes, 75.0% offered ACP. These results are in line with previous literature, describing percentages of persons with advance directives (4.9% and 33%) and PTOs (82%). In the Netherlands, ACP is usually integrated into nursing home care, resulting in a high proportion of residents with dementia having a comfort care goal before death [26].

In German nursing homes significantly fewer wishes for emergency situations were known, ranging from 44.7%- 55.8%. This result is consistent with a previous study, which found that 46% of nursing home residents received ACP in their last months of life [17]. However, the authors suspect a high degree of positive self-selection bias among facilities, making it difficult to generalize to all German nursing homes [17]. We also cannot exclude such selection bias. In around 60–70% of Dutch nursing homes, depending on the situation (e.g. CPR), wishes for residents’ emergency situations were known. This is in line with earlier studies where almost all residents (9 out of 10 residents with dementia) had a comfort care goal at the end of their life and 82% had a PTO [9, 19]. The proportion of Dutch residents with a do-not-hospitalize order increases significantly between nursing home admission and death, from 28 to 76% [27].

These differences could also be due to structural and additionally due to organisational differences in nursing home care between the two countries. In the Netherlands, the health of the residents is regularly discussed in multidisciplinary meetings. Furthermore, there are multiple contact moments between the resident’s relatives and nursing home staff, ensuring frequent medical evaluation and considering the residents and relatives will. These are documented in a treatment plan (PTOs or ADs) [9, 10, 19, 26]. In another study it was been shown, that Dutch ECPs have more contextual knowledge and knowledge of the quality of life of their patients, enabling them to treat based on what they perceived was in the best interest of their patients [28].

German nursing home residents, on the other hand, are usually able to keep their previous GP. Since GPs often have been responsible for their medical care for years, it could be assumed that they potentially know wishes regarding end-of-life care of their patients. However, addressing this issue does not seem to be a frequent part of everyday medical practice.

### End-of-life care

German participants were less likely than Dutch respondents to rate the overall quality of end-of-life care as rather good, and GPs in Germany were considered significantly less well trained for end-of-life care than Dutch physicians. When comparing to existing literature, the German overall rating of end-of-life care is slightly higher than in a previously conducted study (end-of-life care rated as rather good by 64.6%) and perceived training of GPs was in line with a previous study [16]. For the Netherlands, no comparable studies have been published. These different satisfaction levels could be due to the fact that Dutch ECPs have more training in end-of-life care [23, 25]. Cultural differences may also play a role. In the Netherlands, in contrast to Germany, there is more discussion on quality of life versus life-sustaining treatments [9]. It is more common in Dutch nursing homes to refuse potentially distressing life-sustaining treatments. It has been shown that for almost half of the nursing home residents (42.3%) it was decided not to start potentially life-prolonging treatment, and for more than half of the residents (53.7%) this treatment was discontinued [9]. Different attitudes towards the end of life are evident, for example, in the availability of euthanasia in the Netherlands as opposed to Germany [29].

### Measures to improve end-of-life care

In general, the suggested measures to improve end-of-life care in our study were mostly comparable between Germany and the Netherlands, differing only in the most common response. Reflecting that while in Germany staff shortage might have a big impact on end-of-life care, this is not the most pressing issue in Dutch nursing homes.

Respondents from both countries also indicated that better qualification of nursing staff would be a feasible measure to improve end-of-life care. This was also reflected in a previous German and Dutch study [16, 30]. Overall, due to the little amount of suggested measures to improve end-of-life care of Dutch respondents, limited conclusion can be drawn from this and further specific studies are needed to shed light on how to improve end-of-life care.

### Strength and limitations

A strength of our study is, that to our knowledge, this is the first study directly comparing nursing home care in German and Dutch nursing homes with a large and nationwide sample. This makes it possible to directly compare the perspectives of end-of-life care in the two countries. Another strength of this study is, that we offered to answer the questionnaire online and on paper to minimize differences in both countries regarding digitalization.

However, one limitation is that the response from the Netherlands was nearly half than of Germany, possibly affecting generalizability and comparability of these results. One reason could be that in Dutch nursing homes it was often not possible to identify nursing staff managers through manual search to address the questionnaire directly to them. A similarly low Dutch response was described in a previous study, surveying nursing homes in six European countries [31]. Nursing staff managers and facility managers mostly filled out the questionnaire, possibly giving answers that would present their facility more positively. Additionally, the given answers most likely represent the subjective opinion of the person answering the questions. Furthermore, this study merely asked if ACP was offered and did not specify if residents had formal advanced directives, expressed ACP informally, or policy was actually carried out by the treating physician (PTOs). This makes it more difficult to compare to existing literature. However, we asked respondents to estimate how many residents had known wishes for care in emergency situations. This makes it possible to compare known wishes for both countries, irrespective of the existence of a written document.

### Conclusion and implications

We found that in Dutch nursing homes, fewer residents were expected to die in the hospital, more nursing homes offered ACP, more residents’ wishes for emergency situations were known, GPs/ECPs were perceived as better trained in end-of-life care, and significantly more participants rated end-of-life care as rather good than in German nursing homes. These differences could be due to structural differences (ECPs available 24/7 in Dutch nursing homes) and cultural differences (more discussion on quality of life versus life-sustaining treatments and euthanasia being available in the Netherlands). Due to these differences, country-specific approaches are necessary. Overall, more and better-qualified nursing staff and better integration of palliative care would improve the quality of end-of-life care. Future studies are needed to shed light on the specific processes regarding end-of-life care and multidisciplinary collaboration to improve end-of-life care in both countries.

## Supplementary Information

Supplementary Material 1.

## Data Availability

No datasets were generated or analysed during the current study.

## Abbreviations

ACP: Advanced care planning
CHARE-GD I: “Comparison of healthcare structures, processes and outcomes in the Northern German and Dutch cross-border region I”- project
CPR: Cardiopulmonary resuscitation
ECP: Elder care physician
GP: General practitioner
HOMERN: “Hospitalization and emergency department visits of nursing home residents”- project
IQR: Interquartile range
PTO: Physician treatment order
SD: Standard deviation
