# Correction: End-of-life care in German and Dutch nursing homes: a cross-sectional study on nursing home staff’s perspective in 2022

**DOI:** 10.1186/s13690-025-01594-4

**Published:** 2025-04-15

**Authors:** Ann-Kathrin Bauer, Alexander Maximilian Fassmer, Sytse U. Zuidema, Sarah I. M. Janus, Falk Hoffmann

**Affiliations:** 1 Department of Health Service Research, Carl von Ossietzky Universitat Oldenburg, Oldenburg, Germany; 2 Institute of Medical Genetics, Carl von Ossietzky Universitat Oldenburg, Oldenburg, Germany; 3 Department of Primary and Long‑Term Care, University of Groningen, University Medical Center Groningen, Groningen, The Netherlands

Correction: *Arch Public Health***82**, 85 (2024).

10.1186/s13690-024-01316-2.

Following publication of the original article [1], the authors requested to correct the sentence in Study design and data collection section from “The questionnaire (see supplementary file 1) was sent to each selected nursing home in **February** 2022, preferably addressed to the nursing staff manager if the name was available through manual search.” to “The questionnaire (see supplementary file 1) was sent to each selected nursing home in **May** 2022, preferably addressed to the nursing staff manager if the name was available through manual search.”.

The original article [1] has been updated.
