# Supplementary material for: End-of-life care in German and Dutch nursing homes: a cross-sectional study on nursing home staff’s perspective in 2022
Source: Arch Public Health. 2024 Jun 14;82:85. doi: 10.1186/s13690-024-01316-2 (PMC11177492; doi:10.1186/s13690-024-01316-2)
Supplement: Supplementary file 1 — Supplementary Material 1. [file 13690_2024_1316_MOESM1_ESM.pdf]

Medizinische Versorgung in Pflegeheimen: Vergleich zwischen Deutschland und den Niederlanden

CHARE-GD I

Bei Rückfragen wenden Sie sich bitte an:

rijksuniversiteit groningen / Carl von Ossietzky Universität Oldenburg

Dr. Alexander Fassmer | Carl von Ossietzky Universität Oldenburg,
Department für Versorgungsforschung
Ammerländer Heerstr. 114 - 118, 26129 Oldenburg
E-Mail: chare.gd-nursing.homes@uol.de

Dieser Fragebogen richtet sich an das verantwortliche Pflegepersonal in diesem Pflegeheim, z.B. die Pflegedienstleitung oder die Wohnbereichsleitung.
Dieser Fragebogen wird beantwortet für:

☐ das gesamte Pflegeheim ☐ einen Wohnbereich

Medizinische Versorgung und Bedarf

1 **Stimmen Sie der Aussage zu, dass die Bewohner* Ihrer Einrichtung einen hohen medizinischen Versorgungsbedarf haben?**

☐ nein ☐ ja

*Zu Gunsten der besseren Lesbarkeit wird im Fragebogen ausschließlich die männliche Form verwendet (z.B. Bewohner, Hausärzte). Sämtliche Begriffe gelten selbstverständlich im Sinne der Gleichbehandlung grundsätzlich für alle Geschlechter.

2 **Werden die Bewohner Ihrer Einrichtung nur durch eine hausärztliche Praxis (manchmal auch als Heimarzt bezeichnet) versorgt?**

☐ nein ☐ ja

In den folgenden Fragen verwenden wir den Begriff „Hausarzt“ als Oberbegriff für Hausarzt und Heimarzt.

3 **a) Wie viele Hausarztkontakte hat jeder einzelne Bewohner im Durchschnitt pro Jahr? Hierzu zählen sowohl persönliche als auch (video-)telefonische Kontakte.**

ca. .... Kontakte pro Bewohner und Jahr

b) Was schätzen Sie, wie hoch ist der Anteil an Bewohnern Ihrer Einrichtung, die mindestens einmal im Jahr den Bedarf haben für eine Behandlung durch einen

(1) Augenarzt .... % (2) HNO-Arzt .... %

(3) Psychiater .... % (4) Neurologen .... %

c) Denken Sie, dass Hausärzte grundlegende Bedarfe folgender Fachgebiete abdecken können?

	nein, auf keinen Fall	nein, eher nicht	ja, wahrscheinlich schon	ja, auf jeden Fall
Augenheilkunde	☐	☐	☐	☐
Hals-Nasen-Ohren-Heilkunde	☐	☐	☐	☐
Psychiatrie	☐	☐	☐	☐
Neurologie	☐	☐	☐	☐

4 **a) Stimmen Sie der Aussage zu, dass die Bewohner Ihrer Einrichtung einen hohen zahnmedizinischen Versorgungsbedarf haben?**

☐ nein ☐ ja

b) Wie viele verschiedene Zahnärzte versorgen die Bewohner Ihrer Einrichtung (d.h. mindestens ein Kontakt pro Jahr bei mindestens einem Bewohner)?

ca. .... Zahnärzte

c) Welcher Anteil der Bewohner hat mindestens einmal im Jahr Kontakt zu einem Zahnarzt?

.... %

5 **Bei welchem Anteil der Krankenhaustransporte (ambulante Notaufnahmebesuche und stationäre Aufnahmen) aus Ihrer Einrichtung wurde die Entscheidung dazu vom Hausarzt getroffen?**

.... %

6 **Welchen Anteil der Bewohner, die im Krankenhaus stationär behandelt wurden, sieht der Hausarzt innerhalb der ersten Woche nach Entlassung?**

☐ 0-25% ☐ 26-50% ☐ 51-75% ☐ 76-100%

7 **Bitte schätzen Sie die Versorgungssituation insgesamt in Deutschland/den Niederlanden ein. Inwieweit stimmen Sie den folgenden Aussagen zu?**

	gar nicht 0	1	2	3	voll 4
Bewohner von Pflegeheimen werden zu häufig ins Krankenhaus transportiert.	☐	☐	☐	☐	☐
Bei Stürzen von Bewohnern von Pflegeheimen gibt es oft keine Alternative zu einem Transport ins Krankenhaus.	☐	☐	☐	☐	☐
Hausärzte sollten häufiger Besuche in Pflegeheimen durchführen.	☐	☐	☐	☐	☐
Für die medizinische Versorgung in Heimen können telemedizinische Konsile hilfreich sein.	☐	☐	☐	☐	☐

Charakteristika Ihrer Bewohner

8 **Bitte schätzen Sie für Ihre Einrichtung:**

Anteil an Bewohnern mit Demenz:................ %

Anteil an Bewohnern, die ausschließlich bettlägerig sind:................ %

Anteil an Bewohnern mit Dauerkatheter:................ %

Anteil an Bewohnern, die im letzten Jahr mind. einen stationären Krankenhausaufenthalt hatten:................%

Anteil an Bewohnern, die im letzten Jahr mind. einen ambulanten Notaufnahmebesuch hatten:................ %

Anteil an Bewohnern in Kurzzeitpflege:................ %

Versorgung am Lebensende

9 **Bitte schätzen Sie: Bei welchem Anteil Ihrer Bewohner sind die Versorgungswünsche zu folgenden Notfallsituationen bekannt?**

Herz-Lungen-Wiederbelebung .... %

invasive (Tubus-)Beatmung .... %

Behandlung auf Intensivstation .... %

Krankenhaustransport .... %

10 **Bietet Ihre Einrichtung Vorausschauende Versorgungsplanung (Advance Care Planning, ACP) an?**

☐ nein ☐ ja

11 **Wie leicht fällt es Ihnen bzw. den Pflegekräften Ihrer Einrichtung mit den Angehörigen Ihrer Bewohner über das Thema Lebensende zu sprechen?**

☐ sehr leicht ☐ eher leicht ☐ eher schwer ☐ sehr schwer

12 **Bitte schätzen Sie die Versorgungssituation insgesamt in Deutschland/den Niederlanden ein:**

	gar nicht 0	1	2	3	voll 4
Hausärzte sind in der Regel gut für die Versorgung von Bewohnern von Pflegeheimen am Lebensende ausgebildet.	☐	☐	☐	☐	☐
Pflegepersonal der Heime ist in der Regel gut für die Versorgung am Lebensende ausgebildet.	☐	☐	☐	☐	☐

Wie hoch ist der Anteil an Bewohnern von Pflegeheimen, der im Krankenhaus und nicht im Heim verstirbt?

.... %

Wie ist die Versorgung von Bewohnern von Pflegeheimen am Lebensende insgesamt?

☐ eher schlecht ☐ eher gut

→ **Falls Sie mit „eher schlecht“ geantwortet haben, was wäre nach Ihrer Einschätzung die wichtigste Maßnahme, mit der die Versorgung verbessert werden könnte?**

....

....

Angaben zu Ihrer Einrichtung

13 **a) Trägerschaft:** ☐ freigemeinnütziger Träger ☐ privater Träger ☐ kommunaler Träger

b) Anzahl Pflegeplätze: .... Betten

c) Anzahl Wohnbereiche: .... Bereiche

d) In welchem Bundesland/welcher Provinz liegt ihre Einrichtung? ....

e) Wie groß ist der Ort, in dem Ihre Einrichtung liegt?

☐ bis 20.000 Einwohner ☐ über 20.000 bis 100.000 Einwohner ☐ über 100.000 Einwohner

f) Wie weit ist das nächste Krankenhaus mit Notaufnahme von Ihrer Einrichtung entfernt? ca. .... km

g) Ist Ihrer Einrichtung eine der folgenden Versorgungsangebote angegliedert?

☐ Betreutes Wohnen ☐ Tages-/Nachtpflege ☐ Ambulanter Pflegedienst ☐ Hospiz

h) Verfügt Ihre Einrichtung über mindestens einen gerontopsychiatrischen Wohnbereich (für Demenzversorgung etc.)?

☐ nein ☐ ja

i) Gibt es in Ihrer Einrichtung fest angestellte

Ergotherapeuten	☐ nein	☐ ja	Sozialarbeiter	☐ nein	☐ ja
Physiotherapeuten	☐ nein	☐ ja	Psychologen	☐ nein	☐ ja
Logopäden	☐ nein	☐ ja	Ernährungsberater	☐ nein	☐ ja

14 **a) Sind Sie zufrieden mit der hausärztlichen Versorgung der Bewohner Ihrer Einrichtung?**

☐ nein, auf keinen Fall ☐ nein, eher nicht ☐ ja, eher schon ☐ ja, auf jeden Fall

b) Sind Sie zufrieden mit der pflegerischen Personalausstattung Ihrer Einrichtung?

☐ nein, auf keinen Fall ☐ nein, eher nicht ☐ ja, eher schon ☐ ja, auf jeden Fall

15 **a) Verwenden Sie ein festes Notfallprotokoll für den Fall einer akuten Zustandsverschlechterung eines Bewohners?**

☐ nein ☐ ja

b) Verwenden Sie ein festes Überleitungsprotokoll an den Rettungsdienst/ an das Krankenhaus?

☐ nein ☐ ja

16 **Wer richtet/verblistert überwiegend die Medikamente Ihrer Bewohner?**

☐ Ihre Einrichtung selbst ☐ Apotheke

17 **a) Gibt es Therapietiere in Ihrer Einrichtung?**

☐ nein ☐ ja

→ **Wenn ja, welche sind das:**

Hund ☐ Katze ☐ Nagetiere ☐ Vögel ☐ Andere ☐

18 **Nutzen Sie telemedizinische Unterstützung für die Versorgung Ihrer Bewohner?**

☐ nein ☐ ja

Angaben zu Ihrer Person

19 **Alter:** .... Jahre

Geschlecht:

☐ männlich ☐ weiblich ☐ divers

Ihre aktuelle Position:

☐ Pflegedienstleitung ☐ Einrichtungsleitung ☐ Geschäftsführung

☐ Sonstiges, und zwar: ....

Wie lange sind Sie bereits in dieser Position tätig? .... Jahre

Vielen Dank für Ihre Teilnahme!

Medische zorg in verpleeg- en verzorgingshuizen: Vergelijking tussen Duitsland en Nederland

CHARE-GD I

Indien u vragen heeft, kunt u contact opnemen met: Carl von Ossietzky Universität Oldenburg | rijksuniversiteit groningen

Dr. Alexander Fassmer | Carl von Ossietzky Universiteit Oldenburg,
Departement voor onderzoek van gezondheidszorg,
Ammerländer Heerstr. 114 - 118, 26129 Oldenburg, Duitsland
Telefoon: +49 441 798-2766 | E-Mail: chare.gd-nursing.homes@uol.de

Medische zorg en behoeften

1 **Bent u het eens met de stelling dat bewoners van uw instelling grote behoefte hebben aan medische zorg?**

☐ nee ☐ ja

2 **Krijgen de bewoners van uw instelling uitsluitend medische zorg van een specialist ouderengeneeskunde (of een huisarts)?**

☐ nee ☐ ja

In de volgende vragen gebruiken we de term „arts“ als generieke term voor huisarts en specialist ouderengeneeskunde.

3 **a) Hoeveel verschillende Artsen verzorgen ten minste één bewoner in uw instelling?**

ca. .... Artsen

b) Hoeveel contacten met de Arts heeft elke individuele bewoner gemiddeld per jaar?

ca. .... Contacten per bewoner en jaar

c) Wat is het percentage bewoners in uw instelling die minstens één keer per jaar behandeling door een specialist nodig hebben

(1) Oogarts .... % (2) KNO-arts .... %

(3) Psychiater .... % (4) Neuroloog .... %

d) Denkt u dat specialisten ouderengeneeskunde de basisbehoeften van de volgende specialismen kunnen voorzien?

	nee, helemaal niet	nee, waarschijnlijk niet	ja, waarschijnlijk wel	ja, zeker
Oogheelkunde	☐	☐	☐	☐
Keel-Neus-Oor Arts	☐	☐	☐	☐
Psychiatrie	☐	☐	☐	☐
Neurologie	☐	☐	☐	☐

4 **a) Bent u het eens met de stelling dat de bewoners van uw instelling een grote behoefte hebben aan tandheelkundige zorg?**

☐ nee ☐ ja

b) Hoeveel verschillende tandartsen verlenen zorg aan de bewoners van uw instelling (d.w.z. ten minste één contact per jaar met ten minste één bewoner)?

ca. .... Tandartsen

c) Welk percentage van de bewoners heeft ten minste eenmaal per jaar contact met een tandarts?

.... %

5 **Voor welk deel van de ziekenhuisverplaatsingen (ambulante spoedbezoeken en klinische opnames) van uw instelling werd de beslissing genomen door de arts?**

.... %

6 **Welk percentage van de bewoners die in het ziekenhuis opgenomen zijn geweest, ziet de arts in de eerste week na ontslag?**

☐ 0-25% ☐ 26-50% ☐ 51-75% ☐ 76-100%

7 **Geef een cijfer voor de algemene zorgsituatie in Nederland. In hoeverre bent u het eens met de volgende stelling?**

	helemaal niet<br>0	1	2	3	volledig<br>4
Bewoners van zorginstellingen worden te vaak naar het ziekenhuis vervoerd.	☐	☐	☐	☐	☐
Wanneer bewoners van zorginstellingen vallen, is er vaak geen alternatief dan vervoer naar het ziekenhuis.	☐	☐	☐	☐	☐
Artsen zouden vaker hun patienten in een verpleeg- of verzorgingshuis moeten bezoeken.	☐	☐	☐	☐	☐
Telegeneeskundige consulten (via telefoon of videobellen) kunnen nuttig zijn voor medische zorg in een verpleeg- of verzorgingshuis.	☐	☐	☐	☐	☐

Kenmerken van uw bewoners

8 **Geef een schatting voor uw instelling:**

Percentage bewoners met dementie: .... %

Percentage bewoners die uitsluitend bedlegerig zijn: ....%

Percentage bewoners met verblijfskatheters: .... %

Percentage bewoners met ten minste één ziekenhuisopname in het afgelopen jaar: ....%

Percentage bewoners die het afgelopen jaar ten minste één keer naar de eerste hulp zijn geweest: ....%

Percentage bewoners in geriatrische revalidatie (GRZ): .... %

Percentage bewoners in ELV (eerstelijnsverblijf) hoogcomplex: .... %

Percentage bewoners in ELV laagcomplex: .... %

Zorg rond het einde van het leven

9 **Geef een schatting: Voor welk deel van uw bewoners zijn de zorgwensen voor de volgende noodsituaties bekend?**

Cardiopulmonale reanimatie .... %   Invasieve (buis)beademing .... %

Behandeling op de intensive care .... %   Ziekenhuisvervoer .... %

10 **Biedt uw instelling Advance Care Planning (ACP) aan?**

☐ nee   ☐ ja

11 **Hoe makkelijk is het voor u om met familieleden van uw bewoners over het levenseinde te pra-ten?**

☐ zeer moeilijk   ☐ nogal moeilijk   ☐ nogal gemakkelijk   ☐ nogal gemakkelijk

12 **Geef een beoordeling van de algemene zorgsituatie in Nederland:**

	helemaal niet 0	1	2	3	volledig 4
Artsen zijn doorgaans goed opgeleid om zorg aan het einde van het leven te verlenen aan bewoners van zor-ginstellingen.	☐	☐	☐	☐	☐
Verpleeghuispersoneel is meestal goed opgeleid in de zorg rond het levenseinde.	☐	☐	☐	☐	☐

Wat is het percentage bewoners dat in het ziekenhuis overlijdt in plaats van in het verpleeg- of verzorgingshuis?

.... %

Hoe ziet de zorg aan het einde van het leven eruit voor bewoners van zorginstellingen?

☐ nogal slecht   ☐ nogal goed

→ **Als u “nogal slecht” hebt geantwoord, wat zou volgens u dan de belangrijkste maatregel zijn om de zorg te verbeteren?**

....

....

Informatie over uw instelling

13 **a) Bekostiging:** ☐ niet-commerciële   ☐ privé   ☐ gemeente

b) Aantal bedden: .... bedden

c) Aantal woonafdelingen: .... afdelingen

d) In welke provincie is uw instelling gevestigd? ....

e) Hoe groot is de plaats waar uw inrichting zich bevindt?

☐ tot 20.000 inwoners   ☐ meer dan 20.000 tot 100.000 inwoners   ☐ meer dan 100.000 inwoners

f) Hoe ver is het dichtstbijzijnde ziekenhuis met een spoedeisende hulp van uw instelling? ca. .... km

g) Hoe ver is het dichtstbijzijnde ziekenhuis met een spoedeisende hulp van uw instelling?

- ☐ aanleunwoning
- ☐ dag-/nachtbesteding
- ☐ thuiszorg
- ☐ hospice

h) Beschikt uw instelling over ten minste één gerontopsychiatrische woonruimte (voor dementi-ezorg enz.)?

- ☐ nee
- ☐ ja

i) Heeft uw instelling vaste werknemers

ergotherapeuten	☐ nee	☐ ja	maatschappelijk werker	☐ nee	☐ ja
fysiotherapeuten	☐ nee	☐ ja	psychologen	☐ nee	☐ ja
logopedist	☐ nee	☐ ja	dietist	☐ nee	☐ ja

14 **a) Bent u tevreden over de artsenzorg die aan de bewoners van uw instelling wordt verleend?**

- ☐ nee, helemaal niet
- ☐ nee, waarschijnlijk niet
- ☐ ja, eerder
- ☐ ja, zeker

b) Bent u tevreden over het aantal verplegend en verzorgend personeel in uw instelling?

- ☐ nee
- ☐ nogal niet
- ☐ nogal ja
- ☐ ja

15 **a) Gebruikt u een vast noodprotocol in geval van acute verslechtering van de toestand van een bewoner?**

- ☐ nee
- ☐ ja

b) Maakt u gebruik van een vast overdrachtsprotocol naar de ambulancedienst/het ziekenhuis?

- ☐ nee
- ☐ ja

16 **Wie zet de medicatie voor uw bewoners klaar?**

- ☐ Uw instelling zelf
- ☐ Apotheek

17 **a) Zijn er therapiedieren in uw instelling?**

- ☐ nee
- ☐ ja

→ **Zo ja, welke?**

hond ☐ cat ☐ knaagdieren ☐ vogels ☐ andere ☐

18 **Maakt u gebruik van telegeneeskundige ondersteuning voor de verzorging van uw bewoners?**

- ☐ nee
- ☐ ja

Informatie over uzelf

19 **Leeftijd:** .... Jaren

Geslacht:

- ☐ mannelijk
- ☐ vrouwelijk
- ☐ divers

Je huidige functie:

- ☐ Verpleegkundig management/ teamleider zorg
- ☐ Locatie manager
- ☐ Bestuur
- ☐ Andere, namelijk: ....

Hoe lang bent u al werkzaam in deze functie? .... Jaren

Dank u voor uw deelname!
